# Exercise snacking to improve physical function in pre-frail older adult memory clinic patients: a 28-day pilot study

**DOI:** 10.1186/s12877-023-04169-6

**Published:** 2023-08-04

**Authors:** Max J. Western, Tomas Welsh, Kristen Keen, Vanessa Bishop, Oliver J. Perkin

**Affiliations:** 1 Centre for Motivation and Health Behaviour Change, Department for Health, University of Bath, Bath, BA2 7AY UK; 2 Research Institute for Care of Older People, The RICE Centre, Royal United Hospital, Combe Park, Bath, BA1 3NG UK; 3 University of Bristol, Bristol, BS8 1QU UK; 4 Royal United Hospitals Bath NHS Foundation Trust, Bath, BA1 3NG UK; 5 Centre for Nutrition, Exercise and Metabolism, Department for Health, University of Bath, Bath, BA2 7AY UK

**Keywords:** Exercise Snacking, Physical function, Acceptability, Feasibility, Memory

## Abstract

**Background:**

Finding innovative yet feasible ways of preventing physical and cognitive decline in those at risk is a critical global challenge, with exercise being championed as a key precursor to robust health in later life. Exercise snacking, here defined as short bouts of sporadic [muscle-strengthening] exercise, is one such strategy designed to overcome typical participation barriers observed in older adults. This study examined the acceptability of exercise snacking amongst pre-frail older adults and explored the efficacy of this approach in improving physical function.

**Methods:**

In this single group design, 21 pre-frail outpatients with mild-cognitive impairment were recruited from a UK memory clinic. To be eligible, participants were aged ≥ 65-years who scored 3–8 (inclusive) on the short physical performance battery (SPPB) and were not regularly engaging in sport or exercise. Participants completed a 28-day, twice daily, exercise snacking intervention, consisting of five muscle-strengthening exercises, with the aim being to complete as many repetitions as possible of each exercise in a minute. Acceptability of the intervention was measured quantitatively and qualitatively using a survey and topic guide informed by the Theoretical Framework of Acceptability. Pre- and post-intervention physical function was measured using the SPPB, timed up-and-go (TUG), and 60s standing balance and sit-to-stand tests.

**Results:**

Eighteen participants provided follow-up data and showed 85% adherence to the exercise snacking intervention, measured as the proportion of all sessions completed out of a possible 56. Participants rated the intervention as highly acceptable (4.6/5) suggesting it supported their self-efficacy (4.3/5) was enjoyable (4.1/5) and had a low burden (2.1/5). Qualitative findings suggested the ease of use, flexibility of the programme, and perceived effectiveness was important, and particularly useful for non-exercisers. Changes in SPPB score (8(1) vs. 9(3), p < 0.01), TUG (11.32(4.02) vs. 9.18(5.25) seconds, p < 0.01) and in the 60-second sit-to-stand test (17 ± 5 vs. 23 ± 7 repetitions, p < 0.01) were seen between baseline and follow-up.

**Conclusions:**

Exercise snacking is an acceptable and potentially efficacious format of exercise for pre-frail memory clinic attendees who are at heightened risk of falling and frailty. Large scale randomised controlled trials are required to confirm whether exercise snacking is effective in the short and long term.

**ClinicalTrials.gov registration:**

NCT05439252 (30/06/2022)

**Supplementary Information:**

The online version contains supplementary material available at 10.1186/s12877-023-04169-6.

## Background

The prevalence of age associated physical and cognitive decline is markedly rising due to the ageing population and places an enormous strain on health and social care in the UK [1]. One facet of ageing for many older adults is a tendency towards physical frailty, which, among other things, is underpinned by a deterioration in muscle function. The estimated annual loss of muscle size is 0.5-1% per year, with muscle strength lost at 1–5% per year in older age, with this process starting anywhere between 45 and 60 years of age [2, 3]. Eventually the continued loss of muscle strength results in the tasks of daily living becoming too physically strenuous to be managed safely, greatly increasing fall risk and compromising individuals’ ability to live independently [4]. In the UK, muscle weakness in and of itself is estimated to result in excess healthcare costs of £2.5 billion annually [5], with the total annual cost of falling in the UK estimated at £4.4 billion, including £1.1 billion for social care [6]. Indeed, falls are the number one reason for hospitalisation in UK older adults, with at least 1 third of all community-dwelling people aged 65 years or older falling each year [7].

Older people with cognitive impairment are markedly more likely to have reduced physical function and strength, and ultimately a greater fall risk [8, 9]. Up to a third of emergency hospital admissions occur in an older person with dementia, and of these admissions, over half are associated with a fall [10]. Finding ways to engage and support older adults identified as being at increased risk of falls to maintain or even improve their strength and balance will have enormous benefits for both the individuals and wider society. The recently published World Falls Guidelines stress that even in low-risk groups exercise interventions are to be recommended, but it is acknowledged that adherence is particularly challenging [11]. Maintaining strength and balance is key to protecting against a loss of mobility, retaining the confidence and ability to undergo aerobic physical activity and social engagement, and preventing further decline in health [7, 12]. Progressive strength training is undisputedly the most effective countermeasure to age associated declines in muscle mass and function [13].

There is promising evidence that exercise programmes may improve the ability of people with dementia to perform activities of daily living [14]. The UK CMO recommends that older adults undertake exercise sessions (e.g., resistance training) to build muscle strength and balance on two days each week [15]. Recent data however suggests that only 12% of adults aged 65 + and 5% aged 75 + years meet these guidelines [16]. Research suggests that the most common barriers to exercise in older age groups include a perceived lack of time, expertise (i.e., for resistance training), poor access to facilities, low self-confidence for attending gym settings or fear of injury/falling [17–20]. Moreover, cognitive impairment is a potential barrier to engagement and adherence in exercise interventions. As such, there is a need for interventions that are accessible, and to which people can adhere to achieve at least the minimum required dose to promote improvements in functional capacity. Innovative but simple and inexpensive programmes such as homebased ‘exercise-snacking’ have been designed to overcome these barriers, while also providing the functional improvements that would benefit older adults.

Exercise snacking is a mode of exercise that aims to have maximum impact on physical function at minimum ‘cost’ to the patient in terms of time, money and no requirement for specialist exercise facilities or equipment [21, 22]. In other words, the exercise snacks, which are movements designed to increase muscle strength and balance, are to be performed in the home environment over very short periods of time that fit with the current lifestyle of the patient [23]. A novel format of home-based exercise-snacking was designed and refined by researchers at the University of Bath in an attempt to overcome key barriers to engagement of older adults in exercise, by providing a safe, effective, programme that requires no specialist equipment or membership of a gym [18]. Furthermore, the simplicity of the programme and ease of which it is implemented should help empower and boost the self-efficacy of older adults to undertake regular strength and balance exercise [24].

Key to the success of any [new] intervention is that it is centred around the specific needs of the target population [25]. Early indications are that this exercise snacking intervention is safe, beneficial and acceptable to healthy older adults recruited from the general population [21]. However, no research exists examining its potential in outpatient populations who present with increased risk, such as pre-frail older adults who attend a memory clinic. Understanding the perspectives of these target users on the usefulness and ease of implementation is an important first step in ensuring the programme maximises the chance of engaging and benefitting patients. Contemporary research postulates the construct of ‘acceptability’ to be multidimensional, covering elements such as whether a user finds a given intervention to be enjoyable, aligned to their values and identity, easy to understand; not burdensome or displacing of other positive aspects of their life; perceptively effective; and self-efficacy endorsing [26, 27]. Additionally, best practice guidance on the development of health interventions recommends the understanding of the process variables derived to form a theoretical basis and logic model that examine the mechanisms of change or effect [28]. To this end, the present pilot study had two key aims:

1. to examine the acceptability of the exercise snacking intervention guided by the Theoretical Framework of Acceptability (TFA) and identify areas that may need to be improved to optimise it for attendees of a memory clinic.
2. to explore and characterise the potential impact of this intervention on the physical function for this population on outcomes relating to physical function, health, and exercise cognitions.

## Methods

### Study design

This pilot study used a single group, pre-test-post-test design to assess the acceptability homebased ‘exercise snacking’ in older adult patients attending the memory clinic at the Research Institute for the Care of Older People (RICE) in Bath, UK. All participants were asked to undertake 28 days of twice daily exercise snacks, with baseline measures of physical and cognitive function, patient reported health, wellbeing, and psychological process outcomes relating to exercise behaviour, recorded on the day before the intervention and follow-up measures scheduled within 7-days of the final day of the intervention. The primary outcome of intervention acceptability was measured by self-report questionnaire, with participants also invited to participate in a qualitative interview of their experience of the intervention.

### Participants

Outpatients attending the memory clinic at RICE who were identified as being potentially eligible to participate in the present pilot study were invited to participate by RICE gerontology clinicians. Interested individuals were referred to study staff, with clinicians taking no further roll in study procedures. Eligibility requirements were age > 65 years; diagnosed mild cognitive impairment only (Mini-Mental State Examination (MMSE) [29] score ≥ 20); mildly impaired physical function (Short Physical Performance Battery (SPPB) [30] score 3–8 but not scoring zero on any individual component of the test); ability to safely perform 28 consecutive days of homebased exercise snacking; have someone present in the house during exercise snacks who would be capable of calling for help in the event of an emergency; and not currently engaging in regular exercise or participating in another research study with measures or interventions that might interfere with the present study outcomes. Potential participants with contraindications to exercise or co-morbidity preventing participation in exercise (severe breathlessness, pain, psychosis, Parkinson’s, Dementia with Lewy Bodies, or other severe neurological disease), or current musculoskeletal condition that could be made worse through exercise were excluded from participation. The inclusion and exclusion criteria were assessed with a bespoke self-report health screen questionnaire, and eligible participants provided written informed consent. The protocol was approved by the National Health Service (NHS) South West – Frenchay Research Ethics Committee (Ref: 22/SW/0084), conducted in accordance with the Helsinki Declaration of 1975, as revised in 2000, and registered on ClinicalTrials.gov (Identifier: NCT05439252, 30/06/2022). All participants taking part in the study provided written informed consent.

With intervention acceptability as a primary outcome measure, there is a lack of comparable literature to quantitatively estimate a required sample size in this population, given adherence rates to the exercise snacking intervention are yet to be established. As such, for reasons of pragmatism and a fixed duration of study funding, a target of 20 participants to complete follow-up measurements in a three-month testing window was set. Participants who had not adhered to the intervention were included within this target sample size, so long as they would attend follow-up assessment. This approach was taken in an attempt to avoid a withdrawal bias, i.e., only participants who adhered to the intervention returning for follow-up assessment thus positively skewing interpretation of intervention acceptability. A rolling recruitment strategy was employed whereby up to 10 extra participants could be recruited to replace participants who withdrew from the study and were not willing or able to attend follow-up assessments, provided there was sufficient time to enrol and complete follow-up assessments of the replacement participants in the three-month testing period.

### Assessments

Intervention acceptability was assessed by a theoretical framework of acceptability questionnaire on participant experiences of exercise snacking completed at the follow-up assessment only. The seven domains (Affective attitude; Burden; Ethicality; Intervention Coherence; Opportunity Cost; Perceived effectiveness; and Self-efficacy) of the TFA along with overall acceptability were measured using an 8-item questionnaire [27]. An optional qualitative interview to gather further data on the participant’s experience of the exercise snacking intervention was offered to all participants.

During eligibility screening, baseline characteristics were collected (Table 1), and participants completed a health screen questionnaire, the MMSE, and the SPPB. Height (Seca 213 stadiometer, Hamburg, DE) and weight (Seca 799 electronic scales, Hamburg, DE) were measured with participant’s shoes off, and their ability to safely perform exercise snacking was assessed by the trained research psychologist, using a subjective assessment of the participants’ stability and self-reported confidence of completing the assessment.

At baseline and follow-up assessments, participants completed questionnaires on their attitude towards the benefits of exercise using the Multidimensional Outcome Expectancy for Exercise Questionnaire [31], self-confidence for exercise using the Barrier Self-efficacy Scale [32], and satisfaction of the psychological needs for autonomy competence and relatedness in an exercise context using the Psychological need satisfaction for exercise questionnaire [33]. The Patient Health Questionnaire [34] and Generalised Anxiety Disorder Assessment [35] were used to assess mental health, while general health and quality of life was measured using the Short Form Health Survey [36], the Subjective Vitality Scale [37], and the Life Satisfaction Scale [38]. Cognitive function was assessed with the Montreal Cognitive Assessment [39] and self-reported indices of frailty were assessed with the Groningen Frailty Index [40]. The questionnaires were administered by a research psychologist.

Alongside the SPPB, physical function was also assessed by a 60-second sit-to-stand test, in which participants were instructed to complete as many repetitions as possible in 60 s from a hard backed chair with seat height of 45 cm. Participants were permitted to use their arms for balance, but not to use their arms to propel or push themselves out of the chair. To avoid any self-pacing, participants were positioned out of view of a clock and the researcher did not count the repetitions out loud. A single attempt was permitted, and rating of perceived exertion (RPE [41]) was taken immediately after the sit-to-stand test as a secondary measure of test difficulty to capture potential improvements in the event of number of repetitions reaching a physical ceiling for a given participant. As a further test of physical function, the Timed-Up-and-Go (TUG [42],) was attempted twice with the best effort reported. Maximum duration standing balance tests were attempted with feet in semi- and full tandem positions, followed by single leg balance assessment. Balance tests were capped at 60 s, with up to two attempts per position and the best effort reported if the first attempt did not achieve the 60 s of balancing [43].

To characterise habitual physical activity and assess intervention adherence, a wearable physical activity monitor (ActivPAL4, PAL Technologies ltd, Glasgow, UK) was worn on the thigh for the seven consecutive days after the baseline assessment. The device was mounted under an adhesive waterproof dressing so it could be worn continuously, including during water-based activity. Participants were provided with a pre-paid and addressed envelope in which to return the device following seven days of wear.

### Intervention

The exercise snacking intervention consisted of five exercises, each performed for one minute interspersed with one minute of seated rest, with the aim being to complete as many repetitions as possible of each exercise in the given minute. The total length of each exercise ‘snack’ was therefore nine minutes. Participants were instructed to complete the exercises twice a day at home, and only to perform exercise snacks when there was someone else present in the home with capacity to call for help in the event of an emergency. The exercises were sit-to-stand from a chair, seated lateral arm raising to overhead, marching on the spot with high knees (aiming for thigh parallel to the floor), seated arm crossing from arms down by the sides to touching opposite shoulders, and seated calf raises. Participants were provided with a set of written and pictorial instructions on how to complete the exercises along with a logbook in which to record the number of sit-to-stand repetitions completed in each exercise snack, along with a rating of perceived exertion (1–10 scale) for the whole exercise snack. The logbook included space to record minor adverse events or notes from the participant, and clear instruction for reporting serious adverse events to study staff. This logbook was to be returned following the intervention to assess intervention adherence. See Additional file 1 for logbook with exercise instructions.

### Statistical analysis

The primary outcome of acceptability of the exercise snacking intervention was assessed descriptively based on the scores from the TFA questionnaire, and qualitatively using framework analysis [44]. Distribution of continuous data from secondary functional outcome measures (60-second sit-to-stand, TUG, and standing balance tests) were assessed with Shapiro-Wilk normality test. The 60-second sit-to-stand data were normally distributed, so a paired T-test was used to assess differences between baseline and follow-up, and data presented as mean ± standard deviation. The standing balance and TUG data were non-normally distributed, so along with SPPB data (considered to be interval level data in the present study), these were assessed for differences from baseline to follow-up assessment with Wilcoxon matched pairs signed rank test, and data reported as median (IQ range). P-values for physical function outcomes are presented for additional context as to the tests that are of interest for further exploration at larger scale and should not be interpreted as conclusive. For secondary outcome measures, standardised effect sizes were calculated (Cohen’s *d*_*z*_ [45]), and classed as small (0.2), moderate (0.5), and large (0.8) according to Cohen [46]. Secondary outcomes of cognitive function, patient reported health, wellbeing, and psychological process outcomes relating to exercise behaviour are reported descriptively as mean ± standard deviation. Statistical analysis performed using GraphPad Prism 9.5.0 for Windows (GraphPad Software, San Diego, California USA).

## Results

### Participant recruitment and retention

**Fig. 1:**
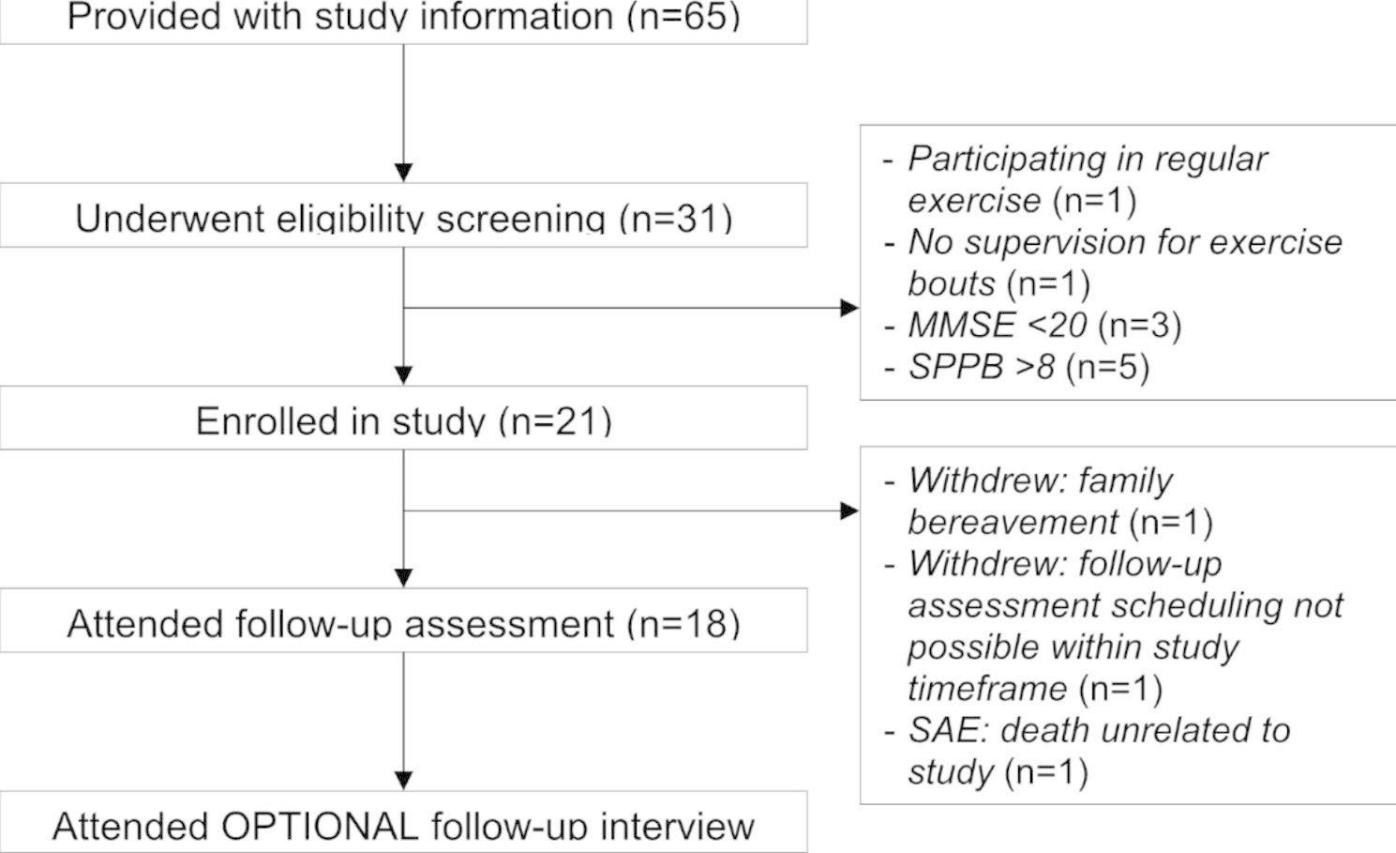
Recruitment flow diagram. *MMSE; Mini-Mental State Examination, SPPB; Short Physical Performance Battery, SAE; Serious Adverse Event.*

Thirty-one participants who attended the memory clinic were screened for inclusion in the present pilot study, of which 21 were included in the study and provided baseline data. The reasons for exclusion at the screening stage are included in the flow diagram in Fig. 1. Baseline characteristics of participants enrolled in the pilot study are displayed in Table 1. Of these, 18 completed the intervention and provided follow-up outcome data (of which one participant attended the follow-up 14 days after the intervention due to illness). Fifteen participants elected to complete the post-study qualitative interview. Reasons for study withdrawal included one participant who experienced a family bereavement, one participant who could not attend follow-up assessment due to logistical issues, and another participant who tragically lost their life owing to an accident unrelated to the study, recorded as an unrelated serious adverse event. Two of the three withdrawals occurred within 28 days of the end of the study timeframe, thus it was not feasible to include replacement participants for these two participants.

**Table 1:** Participant characteristics of N = 21 participants enrolled in pilot study

Characteristic	
Age (years)	78 ± 8
*male*:	*17*
*female*:	*4*
Body mass (kg)	77.7 ± 12.9
BMI (kg/m2)	26 ± 3.9
MMSE Score (/30)	26 ± 3
SPPB	8 (1)
Physical activity (n = 15) *	
*Steps per day*	*4930.1 ± 2556.5*
*Activity score (METs.hour)*	*32.5 ± 1.1*
Ethnicity	
*White British*	20
*Asian British*	1
Marital status	
*Married*:	*17*
*Divorced*:	*4*
Living status	
*Own home*	*21*
Memory clinic diagnosis	
*Mixed dementia*	*3*
*Frontotemporal dementia (semantic)*	*1*
*Alzheimer’s disease*	*7*
*Mild cognitive impairment*	*5*
*Word finding difficulties*	*1*
*Memory loss symptom*	*4*

Data are presented as mean ± standard deviation apart from SPPB, which is presented as median (interquartile range). MMSE; Mini-Mental State Examination, SPPB; Short Physical Performance Battery MET; Metabolic equivalent

* Mean ± SD for those with at least 6 valid days of data (n = 13) are 5175.2 ± 2663.0 steps per day, and 32.6 ± 1.1 MET.hour activity score

### Completeness of data

Of the 18 participants who completed the follow-up assessment, all provided physical function, cognitive function, and health, wellbeing and psychological outcome data across all tests. Sixteen of the 18 participants returned their exercise snacking logbooks, which showed a reported adherence rate of 80% of all exercise snacks completed across the whole group. One participant elected to discontinue the exercise snacking intervention after one week but attended the follow-up assessment and the follow-up interview. Of the 15 participants who attempted 28 days of the intervention, reported adherence was 85%. Half of participants who returned their logbooks completed all exercise snacking sessions over the 28 days. Three accelerometers that were distributed at baseline (n = 21) were misplaced by participants and not returned to the research team, and a further three provided no data owing to technical faults with the device (n = 2) or were removed by the participant. Of the remaining 15 accelerometers, 11 returned complete seven days of valid data, and four had partially complete data (three with six days, and one with three days due to battery fault).

### Acceptability

The quantitative scores indicated an overall acceptability rating from the exercise snacking intervention by completers of the study of 4.6 out of 5. The highest rated aspects of the TFA by study participants were self-efficacy (4.3) and affective response (4.1), followed by ethicality (3.9), coherence (3.8) and effectiveness (3.7). Burden (2.1) and opportunity cost (1.8) were scored low (scores closer to 1 represent a favourable appraisal), demonstrating strong all-round acceptability of the intervention. Similarly, the qualitative interviews indicated a generally positive view of the exercise snacking intervention and insight into some improvements that may enhance the acceptability further (Table 2). In sum, most participants found the programme simple to engage with and fit into their lives, enjoyed participating and recognised the benefits the programme was having on both their physical function and, for some, their mental and cognitive wellbeing, with many declaring confidence to keep up the exercise after the study. For more active participants the programme was considered tedious, while those with more severe cognitive impairment struggled to remember to do the programme every day.

**Table 2:** Qualitative analysis organised using the theoretical framework of acceptability domains, with implications for future implementation

TFA Domain	Theme	Illustrative Quotes	Implications or solutions
*Affective Attitude*	Enjoyable format of exercise	*“Pleased I’ve done it. I quite enjoyed it” (P3473)* *“I enjoyed doing it. I was very grateful that I had someone to help me remember what to do, but I enjoyed throwing my arms around and everything.” (P5150)*	Emphasise enjoyment of exercise via testimonials to help improve buy in and to increase intrinsic motivation
	Surprised by benefit	*“I didn’t know if there wouldn’t be any improvement because they seemed to be so innocuous exercises that I didn’t think they would, but I could feel them as I was doing them…It did, definitely exceed it [my expectations], yes.” (P9075)*	Expect some participants expectations to be low and provide encouragement when initially engaging
	Tedious if already active, or repetitive	*“No. No, I didn’t deliberately stop. But they were tedious and they… and I forgot to do them” (P1667)* *“Most of it is pretty positive, the only negative for me was that it was a little bit boring, it was repetitive.” (P9879)* *“No, I didn’t deliberately stop. But they were tedious” (P1677)*	Screen participants based on existing activity levels or exercise behaviour. Tailor the programme to include progression to avoid tedium.
*Burden*	Easy to fit in and flexible	*“The fact that you could do it when it fitted in with your lifestyle was a good factor…as long as I fitted in the two in the day, that was a good part” (P3473)* *“I felt that the fact that it was structured in that I had to do it twice a day, rather than just do it if it was convenient or whatever, meant that I was more likely to do it. So, I did it virtually all the time.” (P9879)*	Make sure participants are aware of the flexibility and low time commitment to enhance initial engagement.
	Requires energy and motivation	*“If I was tired during the evening as I said, it was difficult to fit in the second one, so perhaps I felt a little bit of negativity then and I think ‘oh twice a day is a bit too much perhaps.” (P3473)* *“I: Was there anything that made it difficult for you to make time to do the exercises? R: Only if I had not slept well the night before” (P8182)*	Suggest that participants find times when they feel most rested or energised to do the snacks Reiterate that they do not need to wait to the evening.
	Memory issues impact compliance	*“Sadly and genuinely I forgot to do the exercises” (P1677)* *“Forgetting, I think I would find it very difficult to do if I didn’t have someone to help me at home, yes. So my memory and my eyesight.” (P5150)*	Find ways to prompt exercise snacks such as environmental cues or support from another person (e.g., spouse, carer).
*Ethicality*	Appropriate for older age	*“Yes, yeah. Winter’s coming we won’t be going out probably so much and then circumstances will change as you get older, we will also start to notice more aches and things.” (P3321)* *“I mean, I’m 74 now. In ten years’ time, probably more need for me to do that type of exercise then ‘cause I’m not gonna find it so easy as I do now.” (P8443)*	Reassure participants of the appropriateness of exercise snacking for older adults as an alternative to traditional exercise.
	Great for non-exercisers	*“Positive. I thought it was good for me, I am not doing enough exercise, I should be moving more, and I thought this was a very good way…I thought this two lots of 10 minutes, it’s not too much so I was able to do it…it seems more manageable than having to go for an hour’s walk every day or something like that.” (P2112)*	Help overcome exercise scepticism by reinforcing the appropriateness of safe and straightforward exercise snacking format
	Physical limitations impact participation	*“I can’t get this arm close to my ear, I can touch my ear with my right arm but because of my shoulder I can’t with the left arm” (P2112)* *“The standing from sitting thing because I’ve got some arthritis in my knees, it made my knees hurt.” (P9879)*	Develop adaptive versions of the core exercises to accommodate differences in physical capability, injury, and pain.
*Intervention Coherence*	Self-monitoring progress is useful	*“The exercises, yeah, I found it helpful just to see how many I’d done the previous day.” (P2112)* *“That was no problem, in fact it was quite an eye-opener to find some days or some parts of the days I was doing less and other days I was doing more of the sitting to standing exercise. But yeah, I had no problem writing it down.” (P3473)* *“Not really. I kept the sheet of paper near my sofa, so that encouraged me and reminded me that I needed to do it, but apart from that, no.”(P4583)*	Emphasise the utility of logging and tracking progress as an intervention function
	Logging sometimes challenging	*“Well, I just got out of the habit of writing it down. I think I mentioned earlier on, it’s no great hardship to write it down but I just got out of the habit of it. I’ll do the exercises and then I’ll forget to write them down or fail to write them down…. So it’s no hardship but if I carry on doing it, as I think I will, I wouldn’t carry on logging it.” (P9879)*	Consider ways to make logging simpler or more habitual and automatic.
	Need for clear, visual instruction	*“Definitely bigger diagrams… these were small, and I couldn’t see them.” (P5150)*	Develop large instruction images and videos to accommodate eyesight issues.
*Opportunity cost*	Sometimes difficult to prioritise	*“No, no, actually I’ve got loads of time, it’s just I’ve got other things to do, that’s all.” (P8084)* *“I had no problem doing the first one. But sometimes it was harder the days, as the day went on and I hadn’t done the second one, it was quite late in the evening and I felt I really had to push myself to do the second one” (P3473)*	Reiterate flexibility in when the snacks can be completed
	Scheduling helped adherence	*“Not too bad because there wasn’t the commitment that I had to do it at a particular time each day, so it was flexible to fit in with what I was actually doing on any given day.” (P4583)* *“None at all. I set two periods of time during the day, morning and late afternoon, and that’s what I did.” (P8443)*	Propose to people who might struggle to fit the exercise snacks in or remember them that planning when to do them could help adherence
	Rationalise study to improve buy in	*“I feel I would like to know what the purpose is, what you were hoping, you medicos, were hoping to glean from the questioning.” (P1677)* *“No, not, no, not really. I probably didn’t feel pressured enough to make sure I did do it absolutely… it’s different when it’s ‘if you don’t do this, it’ll mess up our study’ and I would’ve made more effort.” (P3321)*	Provide a clearer rationale linking exercise and the functional, health and wellbeing benefits.
Perceived effectiveness	Physical function benefits	*“Well, I think they’ve made me think more about staying upright, keeping upright and not slumping and stooping, you know.” (P5150)* *“Yes. Yes, I think in particular the get up, sit down one and the raise your knees to bang your hands ones, I mean they made me, they just made me sort of feel my legs a bit fitter, yeah.” (P7755)* *“And I know that the exercises in question because I could feel them pulling on muscles, were going to be beneficial to those muscles.” (P9879)* *“I was aware that my thigh muscles were a bit stronger when sitting and standing.” (P2112)* *“Without doubt. My knees have got better because of the sitting up, sit up, downs, and I must admit when I first started with the palm exercises my shoulders were stiff as hell and now, they’re far more fluid to say the least, I didn’t realise I never really moved them much before.” (P4583)*	Encourage participants to reflect on the physical feeling post snacking to engage with and appreciate the physical benefit of the programme
	Cognitive and wellbeing benefits	*“I think they might have helped my wellbeing a bit. And it gave me a little bit of structure by having to do them” (P3473)* *“I was hoping that it would give me more strength and mobility n the muscles and the joints that I was using. But I know that physical exercise helps with mental alertness and mental wellbeing” (P9879)* *“R: Yes, they made me think a bit more. I: Okay. What improvement did you expect to see from these exercises? R: A bit more memory, a bit more thinking.” (P2475)*	Reinforce to new participants that the benefits may go beyond impact on cognition, which could help entice people attending a memory clinic or worried about cognitive decline and mental health
	Stimulus too light for some	*“No, because I suppose just by the very nature of exercise you expect it to be demanding and it wasn’t” (P3321)* *“Well, if they did have an effect, I haven’t really noticed it, I just believe it’s good for you.” (P8084)*	Build in progression levels to make the exercise snacks more challenging for those with higher physical function
Self-efficacy	Overcame initial apprehension	*“I was a bit nervous to start with because I didn’t know what it would entail. When I started it was a little bit stressful but then as I got into it and it became a routine, it became easier and easier.” (P4583)*	Reassure participants that the programme is likely to become easier with time if difficult initially
	Social support helpful	*“Well actually my wife did it, did any recording that actually happened, I didn’t. So I can’t really answer that.”(P8084)* *“I’ve even got my wife doing them now.” (P4583)*	Encourage involving others in the programme where available to help confidence
	Would like to continue doing exercise snacking	*“I probably would do once a day but to be honest, I probably wouldn’t do the second one.” (P3473)* *“Absolutely, yeah, I’m going to keep up with… not necessarily twice a day, every day but at least once a day, every day.” (P4583)* *“I feel I should, and I think I would enjoy it and it would be good for me” (P5150)* *“Well it’s introduced me to it, so I probably wouldn’t have started doing it. I mean I’ve threatened to do lots of things, and I did start swimming regularly, but I’m not much of a swimmer, so I tended to let things go. This is relatively easy, and I can do this quickly, so I think I’ll keep it going.” (P9879)*	Provide resources and encouragement to support people to continue after the active intervention period

Where noted in quotes, I = Interviewer; R = respondent

### Exploratory analysis of outcome data

**Fig. 2:**
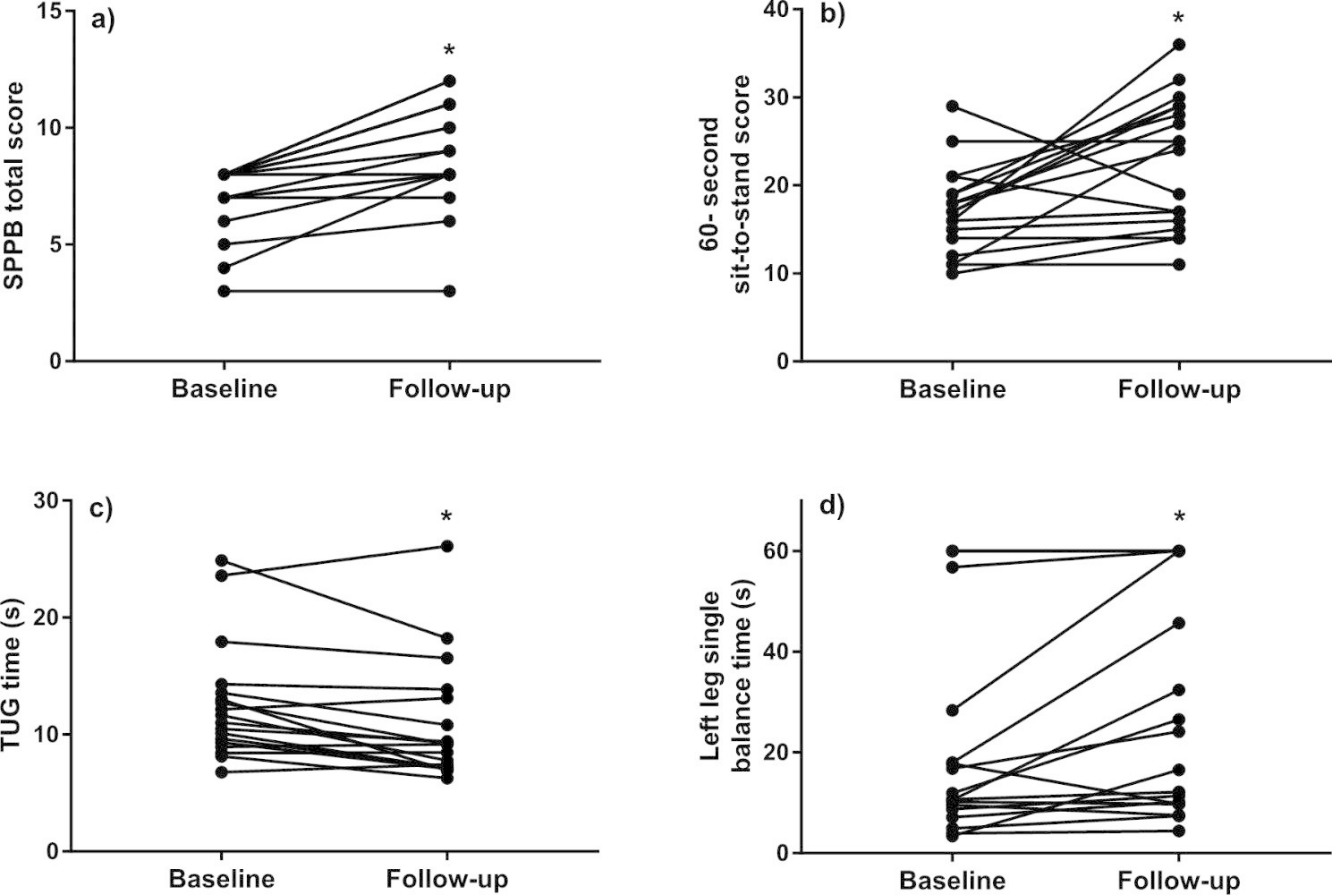
Individual changes in (a) total Short Physical Performance Battery (SPPB) score, (b) 60-second sit-to-stand score, (c) timed up and go (TUG) time following 28-days of exercise snacking, and (d) left leg single balance time (to 60 s). *denotes a significant difference in median (SPPB, TUG, left leg single balance time) or mean (60-second sit-to-stand) from baseline to follow-up (*p* < 0.01)

Changes were observed from baseline to follow-up for total SPPB score (8 (1) vs. 9 (3), *p* < 0.01, *d*_*z*_ = 1.29), TUG (11.32 (4.02) vs. 9.18 (5.25) seconds, *p* < 0.01, *d*_*z*_ = 0.77) and in the 60-second sit-to-stand test (17 ± 5 vs. 23 ± 7 repetitions, *p* < 0.01, *d*_*z*_ = 0.74), with no significant difference in the RPE of the 60-second sit-to-stand tests (11 (3) vs. 12 (3), *p* = 0.36, *d*_*z*_ = 0.22 ). Most participants reached 60-seconds in both baseline and follow-up assessments of semi- and tandem position balance tests, with no differences observed in median balance time between assessments. Single leg standing balance time on the left leg significantly increased (11.27 (16.83) vs. 20.33 (46.56) seconds, *p* < 0.01, *d*_*z*_ = 0.61), with no change observed single leg standing on the right leg (18.15 (51.90) vs. 23.79 (34.78) seconds, *p* = 0.93, *d*_*z*_ = -0.02). Figure 2 displays the individual data for the four physical function outcomes that saw improvements between baseline and follow-up. Table 3 displays the group level baseline and follow-up scores for all health and exercise cognitions outcomes.

**Table 3:** Aggregated descriptive data for secondary outcomes in study completers (n = 18)

	Baseline				Follow-up					
Outcome	Mean	SD	Median	IQR	Mean		SD	Median	IQR	
**Health**										
SF-36 Physical Health	48.5	*8.0*	47.2	*9.7*	46.1		*6.8*	47.6	*12.2*	
SF-36 Mental Health	47.5	*11.9*	54.2	*15.3*	51.3		*12.8*	57.3	*11.0*	
Anxiety	2.0	*2.0*	1.5	*2.8*	1.6		*2.1*	0.5	*2.8*	
Depression	3.5	*3.5*	2.5	*5.0*	3.7		*3.2*	2.5	*4.8*	
Vitality	30.8	*8.7*	34.0	*8.5*	32.4		*6.1*	33.0	*6.0*	
Life Satisfaction	29.0	*4.1*	30.0	*6.0*	27.7		*5.5*	29.5	*6.8*	
General QoL	82.5	*11.1*	85.0	*10.0*	79.4		*8.2*	80.0	*15.0*	
Cognitive Health (MoCA)	23.3	*4.0*	23.5	*4.5*	24.3		*4.0*	25.5	*4.8*	
Groningen Frailty Index	3.9	*2.1*	3.0	*2.8*	3.6		*1.9*	3.5	*3.0*	
**Exercise cognitions**										
Barrier Self-efficacy	70.1	*26.2*	83.8	*31.2*	66.3		*16.3*	67.3	*20.0*	
Outcome exp. Physical	4.5	*0.4*	4.5	*0.6*	4.7		*0.4*	4.7	*0.5*	
Outcome exp. Social	3.3	*1.0*	3.3	*0.8*	3.8		*0.9*	3.8	*1.2*	
Outcome exp. Self	4.1	*0.6*	4.2	*0.6*	4.4		*0.6*	4.5	*1.0*	
Exercise Autonomy	5.4	*0.6*	5.6	*0.6*	5.4		*0.8*	5.9	*1.1*	
Exercise Competence	4.7	*0.9*	4.9	*1.0*	5.1		*0.6*	5.0	*0.9*	
Exercise Relatedness	4.6	*1.4*	5.0	*1.1*	4.4		*1.8*	5.0	*2.5*	

SPPB = short physical performance battery; MoCA = Montreal Cognitive Assessment; SD = standard deviation; IQR = interquartile range

## Discussion

This study provides preliminary evidence that a simple homebased, twice daily exercise snacking programme appears to be acceptable to pre-frail older adult memory clinic attendees. Qualitative data from 15 of the 18 completers suggested that the simplicity of the exercises, and the short, simple to do nature of exercise snacking is a useful feature of this programme, with many non-exercising participants seeing value of continued practice of these exercises. Participants generally found the exercise snacking programme easy to implement, not burdensome, and worthwhile, and for participants who returned follow-up data we observed potential improvements in basic tests of physical function and single leg balance.

Together, these findings offer encouragement for the utility of exercise snacking as an intervention for inactive, older adult, memory clinic attendees, with a large effect size for improvement in the SPPB (*d*_*z*_ = 1.29, and moderate effect sizes for improvements in the TUG and 60-second sit-to-stand (*d*_*z*_ = 0.77 and 0.74 respectively). In a short period, this simple programme has demonstrated improvements in physical function that for a given individual, would present as a positive step towards preventing falls and maintaining independence [7, 12, 47]. Indeed, seven of the 18 participants who completed follow up assessment scored > 9 on the SPPB after the intervention, effectively moving them out the ’frail or pre-frail’ categories on this metric [48]. It should be noted that such physical assessments may be subject to a learning effect [49], and no familiarisation with physical assessments was undertaken prior to baseline, which along with the lack of control group means caution should be exercised when interpreting these change scores and effect sizes.

Self-reported adherence to the exercise snacking intervention was higher than in other exercise studies in people with cognitive impairment (80% of all participants who completed follow up assessment in the present study compared to typical adherence rates around 70%) [50]. Furthermore, while self-reported adherence could be subject to overestimation through social desirability bias, these data may represent an underestimate in adherence owing to participants forgetting to record completed exercise snacks in their logbooks (Table 2). Further studies would do well to deploy appropriate objective measures of capturing adherence, such as the recording of behaviour through technology. Other exercise trials targeting people with mild cognitive impairment have achieved similar improvements in TUG [51], and it is also encouraging that the increase in the sit-to-stand scores were similar to those previously observed implementing a similar exercise snacking intervention in healthy older adults (32% in present study (*d*_*z*_ = 1.29) vs. 31% observed previously (*Hedge’s g* = 1.40) [21]).

Based on our data, exercise snacking may be of particular benefit for those who express common barriers to engagement with more comprehensive exercise provision or formats (such as group-based classes and intensive instructor led interventions), and certainly act as a gateway programme to improve baseline strength and balance and self-efficacy for regular exercise. The process outcomes displayed in Table 3 indicate some of the psychological mechanisms underpinning exercise participation that may potentially be modified by an exercise snacking intervention, namely exercise competence and outcome expectancies [52, 53]. Conversely, scores of other psychological needs and cognitions endorsed by self-determination and social cognitive theory did not change or went down (viz. barrier self-efficacy, autonomy and relatedness), suggesting that additional support may be beneficial for building lasting exercise behaviour change. Of course, given the single-group pilot nature of the present study this exercise snacking programme warrants further testing to evaluate its effectiveness, the processes of change, safety and long-term benefit.

The results indicate several aspects of the protocol and intervention that warrant further consideration prior to full scale testing to improve acceptability, such as the need to develop adaptations of the snacking exercises to make them easier or more challenging as required, or making the logging and performance of exercise snacks simpler and more habitual (Table 2). Furthermore, given challenges in retrieving full data from both exercise logbooks and accelerometers, strategies to aid data collections in cognitively impaired participants requires careful planning and perhaps further patient and public involvement [54].

Consistent with the other evaluations of exercise snacking in general non-patient populations [55], the simple exercise snacking format appears more acceptable to those who do not currently do exercise and ‘tedious’ for those who engage in more physically activity generally. Accordingly, it is proposed that studies evaluating exercise snacking in older adult populations (or practitioners implementing it) target non or low exercisers as those most suited to requiring and receiving such a programme. Consideration of building in progression so that the level of difficulty within the snacking format can be tailored to individual capabilities and exercise experience is also warranted. Secondly, self-monitoring the snacking using logbooks, while considered an important intervention ingredient, was problematic for some memory clinic patients who either forgot to do or log their exercise snacking sessions. Developing innovative yet intuitive ways to prompt the completion and recording of exercise snacks such as the use of digital technologies [56] or aligning to contextual habit-forming cues such as completing an exercise snack after a certain television programme has finished each day [57] may be useful developments of this protocol for those with cognitive impairment. Thirdly, although slight improvements in outcome expectancy was observed from baseline to follow-up, the qualitative acceptability data indicates more effort could be made to improve the rationale by which exercise helps minimise cognitive decline to increase engagement and adherence to the exercise snacking intervention.

There are also several limitations with the study design that warrant consideration. It should be emphasised that the single-group design and lack of a non-exercise control group means that we cannot conclusively state the observed improvements in physical function were a product of the exercise snacking intervention. Whilst the SPPB and TUG are well established with reported reference ranges, the 60-second strength and balance tests used in the present study lack data on their validity and reliability. Furthermore, the present study lacks mechanistic data to explain what physiological adaptations might have contributed to a change in function, for example improved maximum force or power generating capacity [58]. Likewise, the small sample size and analysis of just completers of the intervention means that any statistical results should be interpreted as exploratory and not conclusive. The observed high retention rate, completeness and direction of outcome data and largely positive appraisal of the intervention rate suggest that the study was feasible, acceptable, and potentially efficacious for a pre-frail, mild cognitive impairment population. However, the demographic and health data suggests that the recruited population was fairly homogenous with all participants living in their own home, 20/21 being white British, and having high quality of life and life satisfaction and low depression and anxiety scores (Table 3). Whilst this is likely to be indicative of the population characteristics in the geographical area this study clinic is based, we cannot assume these findings generalise to the wider older adult population in the UK and beyond. The short intervention and lack of further follow-up measurements also means we cannot infer whether any observed changes to the physical function, health or process data, nor adherence to exercise snacking, were sustained in participants.

Considering the findings of this study in light of these limitations, we conclude that the utilisation of a simple exercise snacking intervention could be an acceptable and beneficial way to improve the physical function of pre-frail memory clinic patients and warrants further evaluation to establish the efficacy of this approach. Whilst our data demonstrate an encouraging pattern of overall improvement in functional tests, this is not a robust study design to demonstrate efficacy of the intervention and further research employing a randomised controlled trial study design is required. Nonetheless, these initial findings suggest the exercise snacking format is acceptable, low burden, and engaging in this target population.

### Electronic supplementary material

Below is the link to the electronic supplementary material.

Additional file 1

## Data Availability

The datasets used and/or analysed during the current study are available from the corresponding author on reasonable request.

## List of abbreviations

MET: Metabolic equivalent
MMSE: Mini Mental State Examination
RPE: Rate of Perceived Exertion
RICE: Research Institute for the Care of Older Adults
SPPB: Short Physical Performance Battery
TFA: Theoretical Framework of Acceptability
TUG: Timed Up and Go
