# Supplementary material for: Exercise snacking to improve physical function in pre-frail older adult memory clinic patients: a 28-day pilot study
Source: BMC Geriatr. 2023 Aug 4;23:471. doi: 10.1186/s12877-023-04169-6 (PMC10403822; doi:10.1186/s12877-023-04169-6)
Supplement: Supplementary file 1 — Additional file 1 [file 12877_2023_4169_MOESM1_ESM.pdf]

**Exercise Snacking to Improve Strength and STability: ESISST pilot study**

# Exercise Snacking Logbook

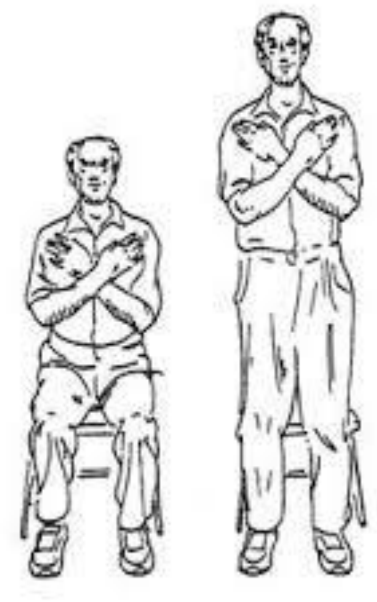

Participant ID: ____________________________

This booklet provides brief guidance on doing exercise snacking, and a logbook for you to record information about two exercise snacks to be completed each day.



Version 1.3 09/03/2022

## Quick guide:

- **Aim to complete two exercise snacks per day- one in the morning and one in the afternoon or evening, for 4 consecutive weeks**

- **There are five exercises in each 'snack'**

- **Attempt one minute of an exercise, followed by one minute of seated rest, before attempting the next exercise**

- **In each minute of exercise, attempt to complete as many full repetitions of the exercise as you can safely**

- **Please perform the sit-to-stand exercise first. The other exercises may be performed in any order after the sit-to-stand**

- **Please record the number of sit-to-stands that you complete each time**

- **Please record how hard you found each exercise snack on the 'Rating of Perceived Exertion (RPE)' scale. See opposite page.**

- **Please add anything else you'd like to record to the comments box, including events such as falls and dizziness during the exercise snacks.**

## *Rating of Perceived Exertion Scale (RPE)*

| | |
|---|---|
| **1** | **No exertion at all** |
| **2** | **Extremely light** |
| **3** | **Very Light** |
| **4** | **Light** |
| **5** | **Moderate** |
| **6** | **Somewhat hard** |
| **7** | **Hard (heavy)** |
| **8** | **Very Hard** |
| **9** | **Extremely hard** |
| **10** | **Maximal exertion** |

- Please use this scale for reference when rating how hard you found each bout of exercise snacking
- This rating should reflect the whole snacking bout, with 1 being no exertion, and 10 being maximum exertion
- Please record this in the ‘RPE’ column of the logbook

# Exercise Snacking- The exercises

## Sit-to-stand:

1. Sit on a firm kitchen chair
2. Rise to a standing position
3. Return to the seated start position at a controlled speed

- *Keep weight even on both feet*
- *You may move your arms for balance if required*
- *To make the exercise easier, you can use your arms to push yourself up from the chair*
- *To add difficulty, you can fold your arms across your chest*

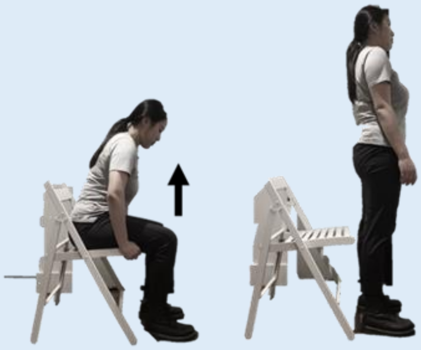

## Seated overhead arm raise:

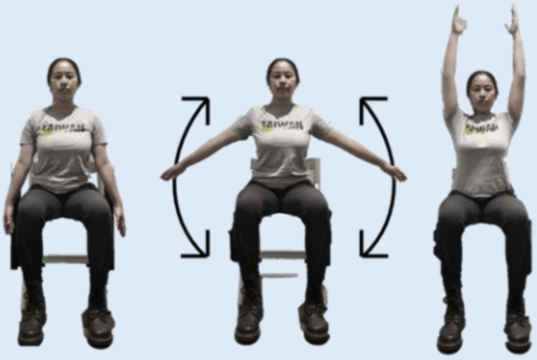

1. Sit up-right on a kitchen chair with arms straight by your side
2. Raise your arms up to the side
3. Reach up until your hands meet above your head
4. Return to the start position at a controlled pace

## Marching on the spot:

1. Stand up-right
2. Hold one or both hands roughly at waist height
3. March on the spot, aiming to touch your thigh to your hands

- *To help balance, you can hold onto something stable like a door frame or table if required*

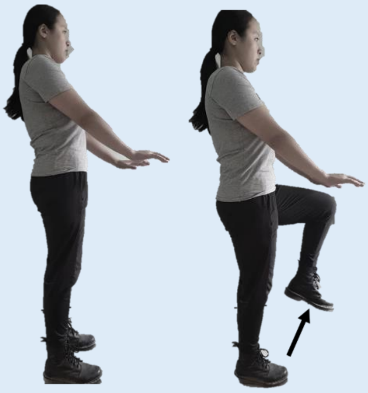

## Seated arm crossing:

1. Sit up-right on a kitchen chair with arms straight by your side
2. Raise your arms straight up in front of you
3. Fold your arms so each hand touches the opposite shoulder
4. Unfold your arms, keeping them straight out in front of you
5. Lower arms down to your side.

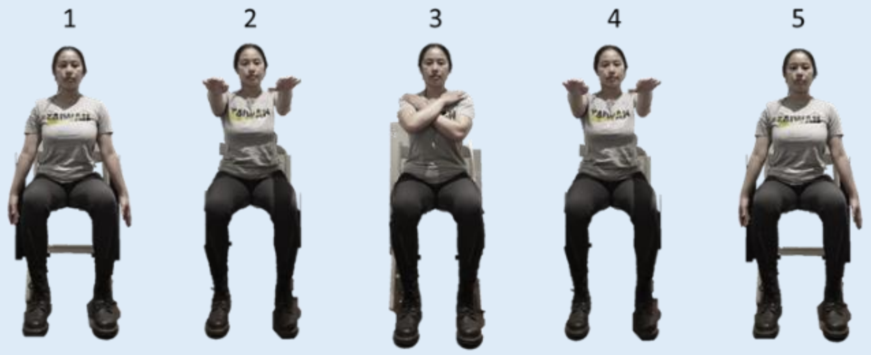


## Seated calf raises:

1. Sit in a chair with your feet flat on the floor
2. Rise up onto your tiptoes as high as you can
3. Return to the start position with feet flat on the floor

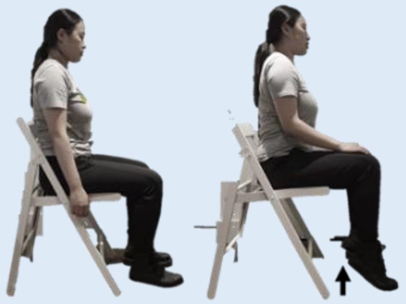

- *Perform the calf raises on both legs at the same time*
- *To add difficulty, you can rest your hands on your knees*

**Please do not perform exercise snacking without someone else in the house who could get help if need.**

- If you feel dizzy whilst exercising, you should stop straight away. If you can, you should sit or lie down until the feeling goes away. Please record this event in the comments section of this logbook and report this to the study researcher at the earliest opportunity.

- In the case of an emergency during a bout of exercise snacking, such as a fall, please do not hesitate in seeking help. Please record this event in the comments section of this logbook and report this to the study researcher at the earliest opportunity.

Contact details of the researcher: 01225 476420

# Week 1

| Day | | Sit-to-stand repetitions | RPE* (1 – 10) | Comments |
|---|---|---|---|---|
| 1 | Snack 1 | | | |
| | Snack 2 | | | |
| 2 | Snack 1 | | | |
| | Snack 2 | | | |
| 3 | Snack 1 | | | |
| | Snack 2 | | | |
| 4 | Snack 1 | | | |
| | Snack 2 | | | |
| 5 | Snack 1 | | | |
| | Snack 2 | | | |
| 6 | Snack 1 | | | |
| | Snack 2 | | | |
| 7 | Snack 1 | | | |
| | Snack 2 | | | |

- **RPE – Rating of Perceived Exertion (see page 3 for full scale).**
    - **1 = no exertion at all, 10 = maximum exertion**

## Week 2– *please post back your activity monitor in week 2*

| Day | | Sit-to-stand repetitions | RPE (1 – 10) | Comments |
|---|---|---|---|---|
| 1 | Snack 1 | | | |
| | Snack 2 | | | |
| 2 | Snack 1 | | | |
| | Snack 2 | | | |
| 3 | Snack 1 | | | |
| | Snack 2 | | | |
| 4 | Snack 1 | | | |
| | Snack 2 | | | |
| 5 | Snack 1 | | | |
| | Snack 2 | | | |
| 6 | Snack 1 | | | |
| | Snack 2 | | | |
| 7 | Snack 1 | | | |
| | Snack 2 | | | |

- **RPE – Rating of Perceived Exertion (see page 3 for full scale).**
    - **1 = no exertion at all, 10 = maximum exertion**

# Week 3

| Day | | Sit-to-stand repetitions | RPE (1 – 10) | Comments |
|---|---|---|---|---|
| 1 | Snack 1 | | | |
| | Snack 2 | | | |
| 2 | Snack 1 | | | |
| | Snack 2 | | | |
| 3 | Snack 1 | | | |
| | Snack 2 | | | |
| 4 | Snack 1 | | | |
| | Snack 2 | | | |
| 5 | Snack 1 | | | |
| | Snack 2 | | | |
| 6 | Snack 1 | | | |
| | Snack 2 | | | |
| 7 | Snack 1 | | | |
| | Snack 2 | | | |

- **RPE – Rating of Perceived Exertion (see page 3 for full scale).**
  - **1 = no exertion at all, 10 = maximum exertion**

## Week 4

| Day | | Sit-to-stand repetitions | RPE (1 – 10) | Comments |
|---|---|---|---|---|
| 1 | Snack 1 | | | |
| | Snack 2 | | | |
| 2 | Snack 1 | | | |
| | Snack 2 | | | |
| 3 | Snack 1 | | | |
| | Snack 2 | | | |
| 4 | Snack 1 | | | |
| | Snack 2 | | | |
| 5 | Snack 1 | | | |
| | Snack 2 | | | |
| 6 | Snack 1 | | | |
| | Snack 2 | | | |
| 7 | Snack 1 | | | |
| | Snack 2 | | | |

- **RPE – Rating of Perceived Exertion (see page 3 for full scale).**
    - **1 = no exertion at all, 10 = maximum exertion**
